# Bell’s Palsy Secondary to COVID-19 Infection

**DOI:** 10.7759/cureus.39037

**Published:** 2023-05-15

**Authors:** Matthew Yu, Anna Virani, Shweta Akhouri

**Affiliations:** 1 Family Medicine, Herbert Wertheim College of Medicine, Florida International University, Miami, USA

**Keywords:** bell’s palsy association, covid-19 pandemic, bell's palsy treatment, covid-19 neurological symptoms, facial nerve palsy, bell`s palsy, covid-19

## Abstract

SARS-CoV-2 (COVID-19) is known to present with a variety of features, with the most common being upper and lower respiratory tract symptoms. However, there are emerging reports of COVID-19 infections with extrapulmonary manifestations, including neurological conditions. We report a case of a patient who presented to his primary care physician with symptoms of Bell's Palsy after recovering from a COVID-19 infection. He was given timely and appropriate treatment that resolved symptoms without residual neurological deficits.

## Introduction

SARS-CoV-2, responsible for the COVID-19 pandemic, has taken a significant toll on healthcare systems worldwide. Having originated in Wuhan, China, in December 2019, COVID-19 is now known to affect many different organ systems, including the cardiac, pulmonary, renal, and central nervous systems [1]. Numerous reports have discussed neurological symptoms, including facial nerve palsy, in COVID-19 patients [2-4]. We report a case of a 52-year-old male, unvaccinated against COVID-19, who presented with right-sided facial paralysis after recovering from a COVID-19 infection in December 2021. The patient was diagnosed with Bell's Palsy and successfully treated with prednisone and acyclovir. As we learn more about the manifestations of COVID-19, primary care physicians play a vital role in encountering patients with various symptoms associated with the virus. It is important to contribute to the current literature on neurological sequelae of COVID-19 infection, especially in the primary care setting.

The etiology of Bell's Palsy (BP) is multifaceted and not completely understood. However, inflammation-mediated macrophage breakdown of neuron myelin sheaths, virus-induced intra-axonal degradation, and fibrosis secondary to ischemia leading to nerve strangulation and compression are all believed to be potential causes of BP [5].

Growing literature reports neurological symptoms associated with COVID-19, including BP, cerebrovascular accidents, and seizures. As per a composite data analysis of 348,088 COVID-19 cases diagnosed between January 1, 2020, and December 31, 2020, 284 cases (0.08%) of new-onset BP were identified [2]. The annual incidence of BP in the general population before the pandemic was estimated at 0.03%. Compared to the vaccinated group, the study found unvaccinated patients had a higher incidence of BP after COVID-19 infection. The mechanism behind the increased incidence of BP in COVID-19 patients is believed to be due to molecular mimicry, as COVID-19 binds to angiotensin-converting enzyme 2 receptors, which are commonly found in neurons [2,4]. COVID-19 spreads to the central nervous system along neuronal synapses and causes an intracranial cytokine storm, resulting in nerve injury and inflammatory demyelination [3].

This article was previously presented as a case report at the 2022 Georgia Academy of Family Physicians (GAFP) Annual Family Medicine CME Weekend on November 10, 2022.

## Case presentation

A 52-year-old male with a past medical history of hypertension presented to the primary care clinic with abrupt right-sided facial paralysis that started three days prior to the visit. He tested positive for COVID-19 four weeks ago with mild cough symptoms. The patient reported a negative COVID-19 test a week before the onset of his facial paralysis. He was unvaccinated against COVID-19, and this was presumed to be his first COVID-19 infection.

He first noticed the paralysis when he could not "squeeze both his eyelids." Upon onset of the facial paralysis, he experienced a mild frontal headache, dizziness, and tingling around his right eye and right upper lip. The patient had never experienced this in the past. The patient denied anosmia, dysgeusia, or other neurological symptoms, including vision/hearing loss, peripheral weakness, and altered sensation.

At presentation, he was afebrile (97.4 degrees F) and mildly hypertensive (133/86 mmHg). His pulse (81/min), respiratory rate (12/min), and oxygen saturation (97%) on room air were all within normal limits. On physical examination, there was complete right-sided hemiparesis of the face, including flattening of the nasolabial fold. He could not raise the right eyebrow or smile on the right side of his face. A sensory exam of the face was normal. The otologic exam showed no rashes or vesicles in the auditory canal, and the tympanic membrane appeared normal without erythema. The ocular exam showed an inability to close the right eyelid. No rashes or vesicles are appreciated on eyelids. Loss of corneal reflex noted in the right eye but preserved in the left eye. The rest of the physical exam, including cardiac and pulmonary, was normal.

His current medications included losartan 100 mg and hydrochlorothiazide 12.5 mg once daily. He consumed alcohol occasionally on special occasions and denied smoking cigarettes. He reported using marijuana in the past but stopped many years ago. He denied any medical history in his family. He denied any recent travel and denied any past herpes simplex virus type 1 (HSV-1) infections.

A clinical diagnosis of BP was made, and he was started on oral prednisone and acyclovir for ten days. He was also advised to use artificial tears in his right eye and a soft eye patch at night to prevent corneal irritation from dry eyes. He was also given a physical therapy referral for facial exercises. At the one-week follow-up visit, he reported that his right-sided facial paralysis resolved shortly after starting medications. On repeat physical exam, right-sided facial drooping had resolved, and he had a symmetric smile. Eyelid weakness had improved significantly, and the patient could close his right eyelid. No new rashes or lesions were noted on the head, ears, eyes, neck, and throat exam. The patient was encouraged to contact the clinic if the symptoms returned.

## Discussion

The most common symptoms associated with a COVID-19 infection are respiratory-related, ranging from fever and cough to shortness of breath [6]. An increasing number of cases have reported neurological complications secondary to the viral infection. A systematic review of 32 papers reporting BP as the only neurological symptom after COVID-19 infection revealed 46 cases from March 2020 to December 2021. Sixty-three percent of patients developed BP after COVID-19 symptoms, whereas 37% presented with BP as the initial manifestation of COVID-19. 72% of these patients showed complete recovery of symptoms, and 23% only showed partial symptom resolution [7]. This case showcases a patient diagnosed with BP a week after recovering from COVID-19.

Other diagnoses on the differential included cerebrovascular accident, Lyme disease, and HSV-1 infection. A cerebrovascular accident was ruled down as the patient did not present with any other focal neurological deficits and, apart from well-controlled hypertension, did not have other risk factors. Lyme disease was also excluded as the patient denied recent travel to the country's Northeast region, exposure to ticks, or new skin rashes/lesions. HSV-1 infection was unlikely as the patient denied past infections and did not present with vesicular rashes or dermatomal pain. BP was ruled up, given the patient's facial neurological symptoms and medical history.

BP is treated with a combination of corticosteroids and antivirals, although there is recent debate on the efficacy of antiviral therapy [8]. The purpose of corticosteroids is to reduce facial nerve inflammation, and that of antivirals is to cover HSV-1, which is believed to play a role in the development of BP. We treated the patient with prednisone and acyclovir for ten days, to which he responded well.

Physical therapy for facial exercises can be a useful adjunct to improve facial function, although no high-quality evidence supports its benefits. However, this approach has little downside or harm [9]. BP is also associated with lagophthalmos, an inability to completely close the eyelids, secondary to facial nerve paralysis [10]. Complications of lagophthalmos include corneal ulceration, perforation, dryness, and infection [11]. The patient was provided artificial tears and a soft eye patch to prevent BP-related corneal complications.

It is also important to point out that numerous patients display long-term sequelae of an acute COVID-19 infection, known as Long Covid. Even patients with mild initial disease may display impaired concentration, headache, sensory disturbances, and depression months later [12]. Studies are underway to examine the rates of Long Covid in patients who were initially treated with antiviral therapy. Our patient, treated with acyclovir promptly, did not report any long-term symptoms upon a follow-up visit to the clinic six months later.

## Conclusions

We are starting to learn more about complications associated with COVID-19 infection. With increasing reports of extrapulmonary and chronic (Long Covid) complications, further studies are warranted to investigate the etiopathogenesis of this virus. Cases of BP post-COVID-19 should be reported to conduct larger studies to investigate the neuroinvasive mechanism of this virus. It is also crucial we report this case because our patient was treated with antiviral (acyclovir) therapy promptly, and he did not develop any chronic symptoms. Primary care physicians need to be aware of this potential sequela of COVID-19 infection to be able to diagnose and treat patients promptly. 
